# Temporomandibular Joint Ankylosis: Long-Term Outcomes with One-Stage Resection and Reconstruction Using Total Joint Alloplastic Replacement—A 20-Year Experience

**DOI:** 10.3390/jcm14134639

**Published:** 2025-06-30

**Authors:** Giovanni Gerbino, Michela Omedè, Elisa Raveggi, Sara Silvestri, Emanuele Zavattero, Guglielmo Ramieri

**Affiliations:** Division of Maxillofacial Surgery, Department of Surgical Sciences, University of Turin, Città della Salute e delle Scienze Hospital, Via Genova 3, 10131 Torino, Italy; giovanni.gerbino@unito.it (G.G.); elisa.raveggi@unito.it (E.R.); sara.silvestri@unito.it (S.S.); emanuele.zavattero@gmail.com (E.Z.); guglielmo.ramieri@unito.it (G.R.)

**Keywords:** temporomandibular ankylosis, temporomandibular joint, prostheses and implants, arthroplasty, replacement, osteoarthritis of the TMJ

## Abstract

**Background/Objectives:** Temporomandibular joint (TMJ) ankylosis, characterized by osseous–fibrous fusion, severely impairs mandibular function. While alloplastic total joint replacement (TJR) is the gold standard, long-term outcomes remain understudied. The aim of this study is to evaluate the long-term esthetic and functional outcomes of prosthetic replacement and to retrospectively analyze our 20-year experience. **Methods:** A retrospective observational study (2003–2024) was conducted at a tertiary referral center in Turin and enrolled patients who underwent alloplastic total joint replacement for TMJ ankylosis. Data collected included demographic variables, etiology, characteristics of the ankylosis, prosthesis type (stock/custom), surgical details, and outcomes (maximal interincisal opening [MIO], pain, quality of life, diet consistency, Helkimo index, complications). **Results:** Among 28 patients (61% female, mean age 51.8 years), etiologies included post-traumatic (39%), osteoarthritis (32%), congenital (25%) and neoplastic (4%) causes. Custom prostheses were used in 57% of cases. Median follow-up was 11.5 years. Significant improvements were observed in MIO (*p* = 0.001), pain level (*p* = 0.001), quality of life (*p* = 0.001), diet score (*p* = 0.002), and Helkimo index (*p* = 0.001). Complications included facial nerve dysfunction (32.1%), salivary leakage (14.3%), and one recurrence (2.2%). **Conclusions:** Alloplstic TJR provides durable functional and esthetic restoration for TMJ ankylosis, with custom prostheses excelling in complex anatomies. Long-term stability (>10 years) supports its role as a definitive solution.

## 1. Introduction

Temporomandibular joint (TMJ) ankylosis is a condition characterized by the osseous–fibrous fusion of joint components, resulting in loss of function. It can arise from various causes, including trauma, infections, inflammatory conditions, and systemic diseases. The primary clinical manifestations include difficulty in performing mandibular movements [1], progressive limitation of mouth opening, facial deformity, and obstructive sleep apnea syndrome (OSAS) [2,3]. Consequently, TMJ ankylosis can lead to mastication, speech, airway and deglutition problems [4], as well as esthetic, oral hygienic, and psychological concerns [5].

The pathogenesis of TMJ ankylosis is not yet fully understood; while several factors have been identified in the literature as contributing to this condition and its recurrence after surgical intervention, the exact mechanisms behind heterotopic bone formation in the TMJ remain unclear [6,7,8].

Particularly challenging is the recurrence of ankylosis, a common issue. Chen et al. identified several risk factors associated with this, including the onset of ankylosis during childhood and temporomandibular joint (TMJ) reconstruction using a coronoid process graft [9].

The incidence of TMJ ankylosis exhibits significant geographic variation, with notably high prevalence reported in regions of Asia and Africa [10,11]. Epidemiological studies indicate a predilection for pediatric and young adult populations, with trauma being the predominant etiology. For instance, a tertiary-center survey in northern India identified 128 ankylosis cases among 1607 pediatric patients (mean age: 12.1 years; 56% female), with trauma accounting for 98% of cases [10]. Similarly, a 9-year Ethiopian review documented 95 cases (56% female; median age: 20–29 years), where 78% were trauma-related [12]. While precise population-level prevalence remains undefined, a Nigerian cohort study found TMJ ankylosis in 1.6% of 3596 mandibular condylar fractures over 16 years [13]. The economic burden of TMJ ankylosis is substantial yet poorly quantified: definitive management requires complex surgery (e.g., gap arthroplasty or joint reconstruction) with significant hospitalization and rehabilitation costs [14]. Indirect costs include long-term loss of productivity and greatly reduced quality of life due to chronic disability. Notably, early surgical release of the ankylosed joint dramatically improves jaw function and patient-reported quality of life, suggesting that delays in treatment would further increase the overall social and economic burden [12,14].

Therapeutic approaches for TMJ ankylosis remain a subject of debate in the literature. Different opinions are reported, depending mainly on surgeon-related factors and age of the patients involved. Several surgical approaches have been documented for treating TMJ ankylosis, like gap arthroplasty, with or without the interposition of biological tissues (autogenous bone, myofascial flaps, cartilages, and fascia lata), or non-biological materials such as acrylic and silastic [15,16]. Other options include reconstruction with autogenous bone graft (costochondral [17], sternoclavicular [18], metatarsal [19], iliac crest [20], fibula [21], and coronoid [22]), coronoidectomy, and total joint replacement with alloplastic prostheses.

Roychoudhury et al. [23], Gerbino et al. [24,25] and Mercuri et al. [26] demonstrated that total alloplastic joint prostheses provide consistent results in terms of morpho-functional results, avoiding donor site morbidity and shortening of operating time.

Prostheses can be stock, preformed, or patient-specific, tailored on patient’s TMJ anatomy.

In 2016, our group published a retrospective study which demonstrated that single stage resection and reconstruction with total alloplastic TMJ reconstruction is an effective and reliable method to re-establish stable long-term mandibular function in ankylotic patients [27].

Moreover, different authors reported that both stock and custom implants allow consistent results [28,29,30]; however, advancements in computer-assisted surgery (CAS), refined planning protocols, and more intuitive software have enhanced the safety and feasibility of custom prostheses, particularly in complex clinical scenarios [31].

However, two critical knowledge gaps persist: no surgical approach has demonstrated consistent efficacy in preventing ankylosis recurrence [32] and long-term outcome data remain notably scarce. To address these limitations, we conducted a 20-year retrospective analysis of alloplastic TMJ reconstructions. This retrospective study aimed to test the hypothesis that alloplastic TMJ reconstruction provides esthetic and functional improvements in ankylosis patients, focusing on prosthesis survival, maximum interincisal opening stability, diet consistency, and patients’ quality of life. Furthermore, we investigated whether these enhancements remained stable over time.

## 2. Material and Methods

In this retrospective observational study, we enrolled patients who underwent alloplastic total joint replacement for TMJ ankylosis from 2003 to 2024 at the Maxillo-Facial surgery division of Città della Salute e della Scienza Hospital in Turin, Italy.

Inclusion criteria were as follows: patients with TMJ bony and fibrous ankylosis (BFA) undergoing alloplastic total joint replacement between 2003 and 2024 in Maxillo-Facial division in “Città della Salute e della Scienza di Torino” Hospital, older than 14 years, with complete pre- and postoperative clinical and imaging records (clinical photos and orthopantomography or computed tomography), and undergone a follow-up period of at least 18 months.

Exclusion criteria were as follows: patients lost at follow-up, decline of general consent, incomplete clinical or radiological data.

The following data were recorded: demographic variables (age, sex, comorbidities), characteristics of the ankylosis (cause, type, grade according to the Sawhney classification [33], duration), concomitant jaw repositioning, type of TMJ prosthesis, previous treatments, and type of postoperative functional physiotherapy protocol. Outcome variables were assessed before surgery and at last follow-up and included: maximum interincisal opening (MIO in mm), pain during mandibular function (NRS from 0 to 10), dietary consistency (in a scale from 0 to 10, in which liquid diet was represented by score 0–3, creamy by 4–6, soft by 7–8, and normal by 9–10), quality of life through Visual Analog Scale (VAS), from 0 to 10 [34], TMJ dysfunction with Helkimo scale, and incidence of relapse. The Helkimo Clinical Dysfunction Index (HCDI) is a simple and quick test used to evaluate subjects affected by temporomandibular disorders, with evaluation of mandibular movements, joint function, pain, and musculature, providing a quick general overview that could be useful at different levels of care [35]. Scoring thresholds classify patients as follows:

- grade 0 (None): no dysfunction (0–1 points);
- grade I (Mild): 2–4 points;
- grade II (Moderate): 5–9 points;
- grade III (Severe): ≥10 points. (Table 1).

Additionally, any complications after surgery were documented. To evaluate the severity of facial paralysis and thus facial nerve function, the House–Brackmann scale was employed, a system which involves a six-point scale with I being normal and VI total, flaccid paralysis [36].

All patients followed a standardized postoperative protocol: a 7-day course of broad-spectrum antibiotic (amoxicillin/clavulanic acid 1000 mg every 8 h) and analgesic therapy (paracetamol 1000 mg every 8 h). A soft diet was prescribed for the first 5 days, with progression to a regular diet as tolerated. Physiotherapy was initiated on postoperative day 1, with gradual intensification based on patient tolerance. Long-term physiotherapy was recommended for a minimum of 1 year, with potential extension depending on individual recovery. The postoperative physiotherapy protocol implemented was as follows:

Phase 1: Early Postoperative (weeks 1–2)

- assisted active hinge opening: 5 repetitions (max 15 mm).
- lateral excursions: 5 repetitions per side (2–3 mm amplitude).
- isometric contractions: light resistance during jaw closing (5-sec hold, 5 repetitions).

Phase 2: Intermediate (weeks 3–6)

- active-assisted range of motions (ROM):
  ○ goldfish exercise: opening to tolerance with tongue stabilized on palate (10 repetitions).
  ○ protrusion/retrusion: controlled forward/backward movements (5 repetitions).
- scar mobilization: gentle circular massage around incision sites (2 min/site).
- postural training:
  ○ chin tucks + shoulder rolls (10 repetitions to correct cervicoscapular alignment).

Phase 3: advanced (weeks 6–12+)

- dietary progression: advanced to solid foods as tolerated.
- resisted training:
  ○ elastic band resistance for opening/closing (3 sets × 8 repetitions).
- functional chewing: soft gum chewing (5 min, 2×/day).
- proprioceptive training:
  ○ mirror biofeedback for symmetrical jaw movement (10 repetitions, 1×/day).

Every session was performed 4–5 times per day for almost 5 min. During hospitalization, patient compliance was assessed daily by the treating physicians. Following discharge, compliance was monitored weekly through scheduled outpatient clinic visits. Someone only performed opening and closure exercises.

Radiographic evaluation consisted of orthopantomography (OPG) and computed tomography (CT) within the first postoperative week to assess the prostheses components position, and the relationship between fixation screws and the inferior alveolar nerve. Follow-up imaging was performed annually and as needed in cases of complications.

### Statistical Analysis

Normality testing was performed using the Kolmogorov–Smirnov test, which confirmed a normal distribution of all quantitative variables (*p* ≤ 0.003 for all datasets).

Descriptive statistics, including mean and standard deviation (SD), were used to summarize the central tendency and dispersion of the data for each parameter both preoperatively (T0) and postoperatively (T1). To compare the mean values of each parameter before and after surgery, a paired *t*-test was applied.

The statistical analysis aimed to compare preoperative and postoperative outcomes across the measured parameters to evaluate the effectiveness of the TMJ replacement surgeries.

To compare the mean values of each parameter before and after surgery within the same group of patients and between the three groups of patients undergone to “No previous surgery”, “1–3 previous surgeries” and “>3 previous surgeries”, a paired *t*-test was applied. This comparative analysis specifically tested the hypothesis that fibrotic scarring and anatomical alterations from previous interventions may attenuate functional improvements. For the mean difference between preoperative and postoperative values, a 95% confidence interval (CI) was calculated to estimate the range within which the true mean difference lies with 95% certainty. A *p*-value was calculated to determine the statistical significance of the observed differences, with a *p*-value of less than 0.05 considered statistically significant, indicating that the observed changes were unlikely to be due to chance.

To quantify treatment efficacy, we calculated Cohen’s d for paired preoperative and postoperative outcomes, with 95% confidence intervals adjusted for multiple comparisons.

## 3. Results

This retrospective observational study included 28 patients—11 males and 17 females—with a mean age at surgery of 51.8 years. The average follow-up period was 10.9 years, with a median of 11.5 years. Six patients had a diagnosis of OSAS. The mean age at the onset of ankylosis was 26 years, and the average time between the onset of the underlying cause and prosthetic replacement was 16.5 years. The diagnoses included in the study were varied: 9 cases of end-stage TMJ osteoarthrosis (32%), 11 cases of post-traumatic ankylosis (39%), 1 case of previous excision of mandibular osteosarcoma (4%) and 4 cases of congenital syndromes (14%) and 3 cases of perinatal pathologies (11%) (Table 2).

**Figure 1:**
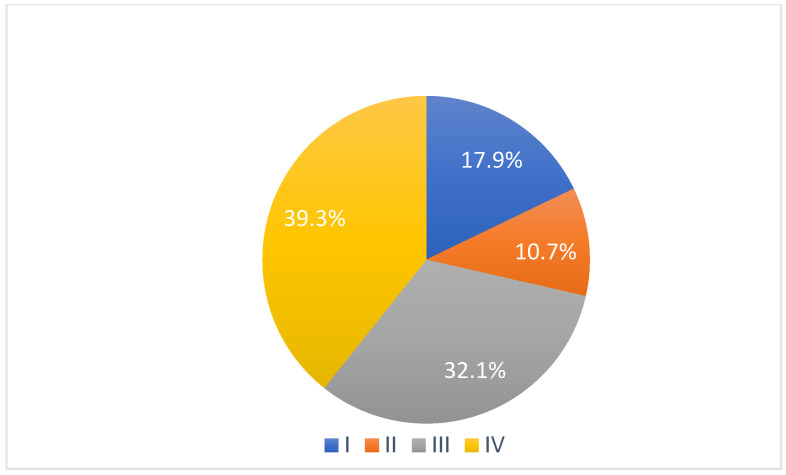
Grade of ankylosis according to Sawhney classification.

According to Sawhney classification, 5 patients were affected by grade I ankylosis (17.9%), 3 by grade II (10.7%), 9 by grade III (32.1%) and 11 by grade IV (39.3%) (Figure 1).

Regarding surgical procedures, 11 unilateral and 17 bilateral joint replacements were performed, summing up to a total of 45 joints being replaced. For 12 patients, Biomet stock prostheses were used, while in 16, custom-made prostheses with cutting guides were utilized (10 Biomet, 1 TMJ-Concept, 5 CADSkills).

Ten patients underwent concomitant jaw repositioning.

**Figure 2:**
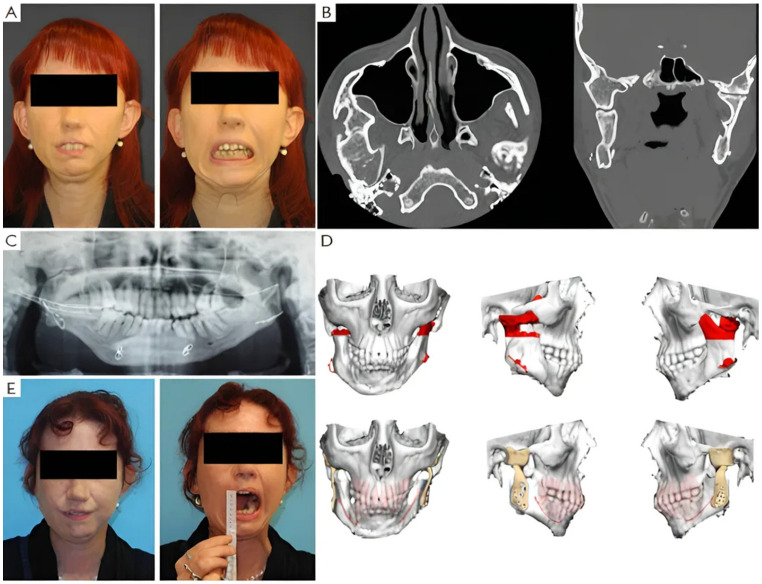
Clinical case of TMJ ankylosis with previous costochondral graft. (A) Clinical frontal preoperative view and preoperative maximum interincisal opening of patient (less than 1 cm). (**B**) Axial and coronal TC scans show bilateral ankylosis. (**C**) Postoperative orthopantomography shows mandible reconstruction with costochondral graft. (**D**) Virtual planning of the resection and virtual planning and design of the prosthesis. (**E**) Postoperative clinical view and postoperative maximum interincisal opening of patient at 1 year follow up (3 cm).

**Figure 3:**
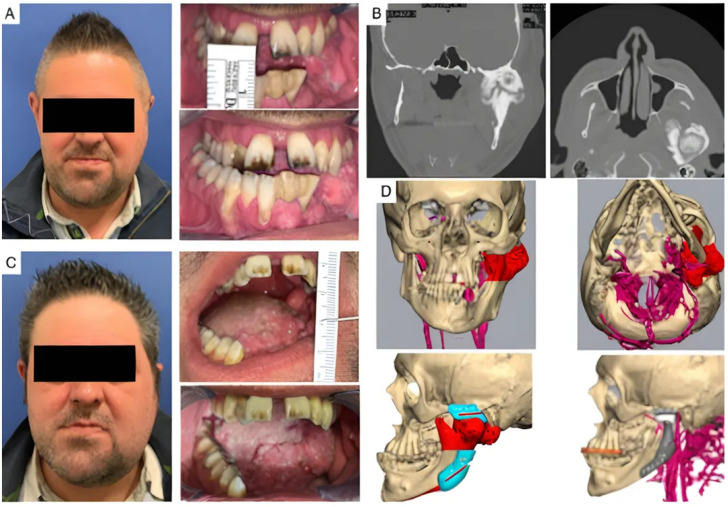
Clinical case of TMJ ankylosis resulted from severe osteoarthrosis. (**A**) Clinical frontal preoperative view and preoperative maximum interincisal opening of patient (less than 1 cm). (**B**) Coronal and axial TC scans show left TMJ ankylosis. (**C**) Postoperative clinical view and postoperative maximum interincisal opening of patient at 1 year follow up (2.5 cm). (**D**) Virtual planning of the resection and virtual planning and design of the prosthesis and of the mandibular repositioning.

**Figure 4:**
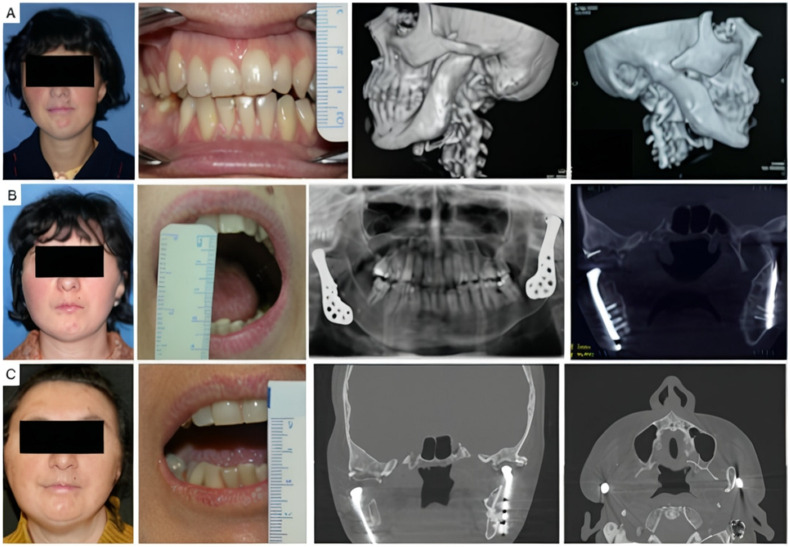
Clinical case of TMJ ankylosis caused by perinatal infection, treated by stock TMJ prostheses. (**A**) Clinical frontal preoperative view, preoperative maximum interincisal opening of patient (less than 1 cm) and preoperative 3D TC scan, that shows bilateral ankylosis. (**B**) Postoperative clinical view, maximum interincisal opening (3 cm) and postoperative radiographs of the patient at 2 years follow up. (**C**) Postoperative clinical view, maximum interincisal opening of patient (1.5 cm) and postoperative radiographs at 17 years follow up.

Four patients had not undergone previous surgical treatments, while 21 had undergone one to three prior surgeries. Three patients had more than three interventions. Of these, 13 underwent coronoidectomy, 10 underwent arthroplasty without material interposition, 7 experienced maxillo-facial fractures osteosynthesis, 2 had condylectomies, 2 mandibular distraction, 4 costochondral grafts, 1 an iliac crest graft, 2 arthroplasty with silastic interposition, 1 dermal fat interposition, and 2 had previous total joint replacements with stock prostheses. Three patients’ cases are illustrated in Figure 2, Figure 3 and Figure 4.

Significant postoperative improvements were observed. The maximum interincisal opening (MIO) increased from 10.4 mm to 29.7 mm, with a mean difference of 19.3 mm, which was statistically significant (*p* < 0.001). Pain levels, measured on a Numerical Rating Scale (NRS), decreased from 6.0 to 1.3, with a mean reduction of 4.7 points (*p* < 0.001). The diet score improved from 5.9 to 9.1, and the quality of life (QoL) score increased from 3.8 to 8.6, both changes being statistically significant (*p* < 0.001). Additionally, the Helkimo Index, which measures TMJ dysfunction, decreased significantly from 14.2 to 2.0 (*p* < 0.001) (Table 3).

Pre- and postoperative measurements of maximum interincisal opening (MIO), temporomandibular joint (TMJ) pain, diet consistency, quality of life (QoL), and Helkimo Index scores were compared across three patient groups: those with no prior surgery, one to three prior surgeries, and more than three prior surgeries.

In the “No previous surgery” group (n = 4), three variables showed statistically significant improvements after treatment: MIO increased from 6.8 mm to 35 mm (*p* = 0.001), QoL scores improved from 4.3 to 9 (*p* = 0.005), and the Helkimo Index decreased from 10 to 1.5 (*p* = 0.016). (Table 4).

These results were observed in the “1–3 previous surgeries group” (n = 21): all measured parameters demonstrated statistically significant improvements (TMJ pain: *p*-value < 0.001; diet consistency: *p*-value < 0.001; MIO: *p*-value < 0. 001; quality of life: *p*-value 0.006; Helkimo score: *p*-value < 0.001). Specifically, patients exhibited a mean increase of 16.9 mm in MIO, a 5-point reduction in TMJ pain, a 3.5-point improvement in diet consistency, a 4.9-point gain in QoL scores, and a 12.7-point decrease in Helkimo Index values (Table 5).

The “>3 previous surgeries” group (n = 3) also displayed statistical significant improvements, though the small sample size limits statistical reliability. TMJ pain scores dropped from 8 to 1.3 (*p* = 0.01), the Helkimo Index improved from 15.7 to 3.3 (*p* = 0.026), MIO increased from 5 mm to 33 mm (*p* = 0.008), and QoL scores rose from 3.3 to 9 (*p* = 0.003). Diet consistency showed a numerical improvement (6.7 to 9.3), but this did not reach statistical significance (*p* = 0.208) (Table 6). The sample size, however, is too small for reliable inference.

To quantify treatment efficacy, we calculated Cohen’s d for paired pre- and post-surgical outcomes on total sample and in the three groups, with 95% confidence intervals adjusted for multiple comparisons; results are summarized in Table 7 and in Table 8.

Postoperative complications included: one case of recurrence (0,02%) on 45 temporomandibular joint replacements, caused by heterotopic bone formation, four cases of salivary leakage and one case of prosthesis infection. Facial nerve function could be assessed in twenty-five patients, as three patients had pre-existing facial paralysis. Four (16%) showed permanent facial nerve dysfunction; three had mild motor dysfunction according to the House–Brackmann scale (grade II), affecting mainly the upper, mid and lower face, while two reported moderate or moderately severe dysfunction (grade IV–V). Five patients (20%) experienced facial nerve weakness that resolved within 6 months.

Two patients experienced hardware malpositioning requiring surgical revision. The first case involved a stock prosthesis with postoperative malocclusion, which necessitated hardware repositioning four days after the initial surgery. The second case concerned a custom-made prosthesis with jaw repositioning, in which the planned occlusal relationship could not be achieved due to excessive soft tissue tension in a patient with a history of multiple surgeries. The surgical plan was revised, new devices were fabricated, and the hardware was replaced, resulting in a less ambitious yet stable repositioning of the maxillomandibular complex. Maximum interincisal opening (MIO) was 30 mm.

The relapse case underwent reoperation, including intraoperative hardware removal, heterotopic bone resection, replacement of the original hardware, and free fat graft placement. The patient has remained asymptomatic with no recurrence after a five-year follow-up.

The infection case involved an early postoperative infection, managed with prompt surgical debridement, retention of the prosthesis following the Wolford protocol [38], and prolonged intravenous antibiotic therapy. The infection resolved, and the patient has shown good clinical outcomes at five-year follow-up.

A total of 16 patients followed a mobilization protocol with specific mandibular exercises, while 12 patients followed a traditional opening and closing exercise regimen. No significant difference in MIO was observed between the two mobilization protocols (*p* = 0.272).

## 4. Discussion

Management of TMJ bony ankylosis remains a significant clinical challenge. A great variety of techniques have been described, like gap arthroplasty with or without interposition of material [39], with better outcomes for interpositional arthroplasty in terms of mouth opening and re-ankylosis [40], and reconstruction arthroplasty with autogenous and alloplastic material [41]. However, no single strategy has been universally accepted [6,7].

Re-ankylosis represents a frequently observed complication, with no currently available technique demonstrating consistent efficacy in preventing recurrence; predisposing factors include costochondral grafts, inadequate surgery, low patient compliance for physiotherapy, high remodeling and bone turnover in children and fibrosis of longstanding inactive muscles involved in mastication [9,42].

Trauma and infections are the most common etiologies of ankylosis; however, cases have also been reported involving systemic diseases, TMJ tumor resections, previous TMJ surgical interventions, and failed alloplastic TMJ reconstructions [43]. In this study, trauma accounted for 11 cases of ankylosis, a complication frequently resulting from suboptimal management of condylar fractures, particularly those involving prolonged immobilization. Four cases were congenital, associated with Klippel–Feil, Treacher–Collins, and Goldenhar syndromes, while three were of perinatal origin secondary to local infections (otogenic, dermatogenic, or odontogenic with subsequent osteomyelitis). Additionally, we identified nine cases of end-stage temporomandibular joint (TMJ) osteoarthrosis and one case following mandibular osteosarcoma resection.

The study conducted at the University of Torino provides a comprehensive evaluation of the long-term clinical outcomes and complications associated with temporomandibular joint (TMJ) prosthetic replacement.

All patients in this study were treated with single-stage resection of the ankylotic block, followed by reconstruction with either stock or custom-made total TMJ prostheses.

Mercuri [44], in 2012, proposed a two-stage surgical protocol for ankylosis and re-ankylosis management: initial resection of the ankylotic mass with interpositional spacer placement to restore mandibular mobility, followed by definitive joint reconstruction.

However, a two-stage approach requires multiple surgeries, which increases the risk of scarring and facial nerve damage.

The use of a fully digital workflow, with CAD-CAM cutting and positioning guide [27], may eliminate the need for a two-stage procedure and enable reliable surgical outcomes, as demonstrated by Haq [45] and Gerbino.

In our series, ten patients underwent concomitant jaw repositioning, in order to correct skeletal deformity associated OSAS, occlusal, and esthetic features.

Concomitant TMJ replacement and orthognathic surgery allow us to reduce both treatment time, surgical risks and increase the overall success of treatment [18,22,40,46].

The observed improvement in maximum interincisal opening (MIO from 10.4 mm preoperatively to 29.7 postoperatively) is a significant outcome and aligns with findings from other studies. For instance, Wolford et al. [47] reported an average postoperative MIO of 32 mm in patients who underwent total joint replacement with custom-made prostheses, highlighting the effectiveness of these devices in restoring jaw function.

In patients with ankylosis, the elasticity of periarticular soft tissues is significantly diminished, particularly in those who have undergone multiple surgeries or have long-standing ankylosis. Consequently, restoring normal jaw opening under these conditions is unfeasible. Thus, achieving sustained mobility without a relapse of mandibular opening limitations could be considered a successful outcome [29].

Regarding pain reduction, this study reports a decrease in mean pain levels from 6.0 to 1.3; Mercuri et al. and Sidebottom et al. [8,48] similarly noted substantial pain relief following TMJ replacement, with long-term studies demonstrating sustained low pain levels.

Quality of life (QoL) scores improved from 3.8 to 8.6, consistent with outcomes reported by Driemel et al. [49] and Beret et al. [50], who found significant improvements in patients’ quality of life following TMJ replacement.

The results of the Global Aesthetic Improvement Scale (GAIS) after TMJ prosthetic surgery revealed a wide range of outcomes, highlighting the complexity of achieving both functional and esthetic success in such cases. Gerbino et al. [31] emphasized that, while functional restoration is the primary goal of TMJ prosthetic replacement, achieving satisfactory esthetic outcomes can be difficult, especially in cases with significant preoperative asymmetry or deformities. In this study, 35.7% of patients reported a large improvement in their esthetic appearance, 10.7% reported a slight improvement and 53.7% of patients reported no significant differences in appearance. No one reported worsening. These findings must be interpreted with caution, given the clinical complexity of this patient cohort. The majority presented with long-standing ankylosis—often since childhood—rendering even marginal functional gains clinically significant.

A notable finding is the difference in outcomes between custom-made and stock prostheses. Patients with custom prostheses generally reported better outcomes, with 50% experiencing large improvements in quality of life, compared to only 16.7% in the stock prosthesis group. Custom prostheses are more anatomically tailored on the patient and more precise in restoring face contours, leading to better functional and esthetic outcomes. The literature supports the superiority of custom prostheses in TMJ surgery: Gerbino et al. [31] have demonstrated that the utilization of custom-made prostheses in conjunction with virtual surgical planning provides superior precision and enhanced predictability when compared to conventional stock devices, especially in difficult cases and when jaw repositioning is deemed to be necessary. The precise fit of computer-generated custom implants will reduce the chance of micromovement underloading, with less stress on fixation systems, thus increasing the lifespan of the implants themselves.

In our study, we evaluated two protocols of physiotherapy for rehabilitation: mouth opening and closure or specific exercises [51], which include also protrusion and lateral deviation movement of the mandible; no significant differences were found in terms of MIO between the two groups (*p* = 0.272). However, it is crucial for patients to begin mobilizing the mandible immediately after surgery and to continue exercises long-term. This is consistent with findings from Ravinder et al. [52], who demonstrated that physiotherapy is essential to optimize postoperative outcomes and minimize adverse events such as re-ankylosis.

Aagaard et al., Gerbino et al., and Haq et al. have evidenced that virtual surgical planning, facilitated by advanced software systems, significantly improves the efficiency of patient-specific total temporomandibular joint (TMJ) reconstruction [29,31,45]. Computer-generated cutting guides enable accurate translation of virtual surgical planning to the operative site. Patient-specific implant components are designed using computer-aided design/computer-aided manufacturing (CAD/CAM) technology to precisely match individual anatomical requirements.

An important issue in TMJ treatment is the risk of re-ankylosis: Chen, Yadav et al. identified key risk factors for ankylosis recurrence [9], including its onset in childhood and TMJ reconstruction with a coronoid process graft, and, with a retrospective study, defined a protocol to minimize this risk [9,53]. They found that certain factors contribute to this: (1) complete removal of the ankylotic mass; (2) use of a piezoelectric scalpel, with copious irrigation to remove bone chips and slurry; (3) less trauma to the local tissue; (4) osteotomy design parallel and inferior osteotomy at the narrowest part, which mostly corresponds to the condylar neck; (5) performance of a coronoidectomy (if mouth opening is <30 mm), (6) fat interposition; (7) aggressive physiotherapy; and (8) use of a vacuum drain.

Fat grafting has been studied by several authors, including Roychoudhury et al., Mercuri et al. and Wolford et al. [8,54,55], who demonstrated that total joint replacement with stock prostheses, along with fat grafting around the joint, provides adequate and lasting mouth opening while reducing the chances of reankylosis.

Key factors in preventing re-ankylosis in our treatment protocol are the following:

(1) complete resection of the ankylotic mass, (2) coronoidectomy, and (3) single-stage total joint resection and reconstruction using alloplastic prostheses. This comprehensive approach facilitates immediate postoperative mobilization and enables early initiation of intensive mandibular rehabilitation therapy.

The main limitation of this study is the relatively small sample; thus the median follow up is consistent. Further large-scale, multicentric studies are required to assess a broader population and enhance control over variables.

## 5. Conclusions

Evidence supports single-stage alloplastic total joint reconstruction as a durable solution for restoring mandibular function in TMJ ankylosis patients, with stable long-term outcomes, a reduction in TMJ pain, functional improvement, and enhanced quality of life, as demonstrated by large effect sizes obtained with Cohen’s d. The low incidence of relapse (1/45 TMJ replaced) further underscores its reliability. Custom-made prostheses with cutting guides are particularly advantageous in congenital and perinatal ankylosis cases, where severe anatomical disruption demands patient-specific solutions. While this study highlights the potential benefits of custom prostheses, these conclusions require cautious interpretation, especially concerning the alleged superiority of custom-made prostheses compared to stock ones, due to the absence of direct statistical comparisons between prosthesis types in matched cohorts. The retrospective design limits control over confounding variables, including heterogeneity in surgical indications, ankylosis severity, and rehabilitation protocols. Additionally, the lack of a non-surgical control group precludes definitive assessment of whether observed improvements exceed natural disease progression. Future prospective randomized controlled trials are needed to validate these findings.

## Figures and Tables

**Table 1:** Helkimo Clinical Dysfunction Index (HCDI).

Domain	Criteria	Score
Mandibular mobility	MIO > 40 mm, excursions > 7 mm	0
	MIO > 30 mm, excursions > 3 mm	1
	MIO ≤ 30 mm, excursions ≤ 3 mm	5
TMJ Dysfunction	No sounds/deviation on opening	0
	Sounds and/or deviation > 2 mm	1
	Locking and/or luxation	5
Muscle pain	No muscle pain	0
	Pain on palpation at 1–3 sites	1
	Pain on palpation at 4+ sites	5
TMJ pain	No tenderness to palpation	0
	Lateral (superficial) TMJ pain	**1**
	Posterior (deep) TMJ pain	**5**

**Table 2:** Causes of ankylosis.

Cause	Number	Percentage
Inflammation	9	32%
Trauma	11	39%
Congenital syndromes	4	14%
Perinatal infections	3	11%
Neoplasm	1	4%

**Table 3:** Mean values of each parameter before and after surgery within the same group of patients.

BFA n = 28	T0 Mean (SD)	T1 Mean (SD)	Mean Difference (IC)	*p*
MIO (mm)	10.4 (6.8)	29.7 (8.1)	19.3 (15.6; 23.1)	<0.001
PAIN (NRS)	6.0 (3.4)	1.3 (2.0)	−4.7 (−6.1, −3.3)	<0.001
Diet score	5.9 (2.5)	9.1 (1.7)	3.2 (2.3, 4.2)	<0.001
VAS QoL	3.8 (1.5)	8.6 (1.4)	4.8 (4.0; 5.6)	<0.001
HELKIMO INDEX	14.2 (5.8)	2.0 (2.0)	−12.2 (−14.3, −10.0)	<0.001

**Table 4:** Mean values of each parameter before and after surgery within the group of patients never operated.

0 Prior Surgeries (n = 4)	T0	T1	Mean Difference (IC)	*p*
	Mean (SD)	Mean (SD)		
MIO (mm)	6.8 (5.2)	35 (5)	28.2 (21, 35.5)	0.001
PAIN (NRS)	4.8 (3.2)	1.8 (2.1)	−3 (−4.9, 10.9)	0.314
Diet score	7.5 (2.1)	9.5 (0.6)	2 (−1.9, 5.9)	0.201
VAS QoL	4.3 (1)	9 (0.9)	4.7 (2.7, 6.7)	0.005
HELKIMO INDEX	10 (3.2)	1.5 (0.6)	−8.5 (−13.9, −3.1)	0.016

**Table 5:** Mean values of each parameter before and after surgery within the group of patients operated 1–3 times before.

1–3 Prior Surgeries (n = 21)	T0	T1	Mean Difference (IC)	*p*
	Mean (SD)	Mean (SD)		
MIO (mm)	11.5 (7.3)	28.4 (8.6)	16.9 (12.6, 21.3)	<0.001
PAIN (NRS)	6.4 (3.3)	1.4 (2.1)	−5 (−6.5, −3.5)	<0.001
Diet score	5.6 (2.6)	9.1 (1.9)	3.5 (2.3, 4.6)	<0.001
VAS QoL	3.7 (1.7)	8.7 (0.9)	4.9 (4.1, 5.8)	0.006
HELKIMO INDEX	14.7 (6.4)	2.0 (1.7)	−12,7 (−15.5, −9.9)	<0.001

**Table 6:** Mean values of each parameter before and after surgery within the group of patients operated >3 times before.

>3 Prior Surgeries (n = 3)	T0	T1	Mean Difference (IC)	*p*
	Mean (SD)	Mean (SD)		
MIO (mm)	5 (1)	33 (4.6)	28 (17.2, 38,8)	0.008
PAIN (NRS)	8 (2)	1.3 (2.3)	−6.7 (−9.5, −3.8)	0.01
Diet score	6.7 (1.5)	9.3 (1.1)	2.6 (−3.6, 8.9)	0.208
VAS QoL	3.3 (0.6)	9 (1)	5.7 (4.2, 7.1)	0.003
HELKIMO INDEX	15.7 (2.3)	3.3 (3)	−12.3 (−21, −3.6)	0.026

**Table 7:** Effect size estimates (Cohen’s d with 95% CIs) for pre-to-post surgical improvements in the total cohort. The effect size interpretation is the following: 0.1 = small; 0.3 = medium; 0.7 = large [37].

Variable	Cohen’s d	95% CI	Interpretation
NRS	1.92	[1.32, 2.52]	Large Effect
Diet	1.05	[0.55, 1.55]	Large Effect
MIO	1.78	[1.20, 2.36]	Large Effect
Helkimo	2.31	[1.65, 2.97]	Large Effect
VAS	0.89	[0.40, 1.38]	Large Effect

**Table 8:** Effect size estimates (Cohen’s d with 95% CIs) for pre-to-post surgical improvements in the three groups stratified by prior surgical history (Group 1: no previous surgeries, Group 2: 1–3 prior surgeries, and Group 3 > 3 prior surgeries). The effect size interpretation is the following: 0.1 = small; 0.3 = medium; 0.7 = large [37].

Variable	Group 1 (n = 4)	Group 2 (n = 21)	Group 3 (n = 3)
NRS	2.10 [1.01, 3.19]	1.85 [1.21, 2.49]	2.40 [0.01, 4.79]
Diet	1.25 [0.20, 2.30]	1.02 [0.50, 1.54]	0.90 [−0.60, 2.40]
MIO	2.80 [1.40, 4.20]	1.65 [1.05, 2.25]	3.10 [1.20, 5.00]
Helkimo	2.50 [1.25, 3.75]	2.30 [1.60, 3.00]	1.80 [0.10, 3.50]
VAS	1.10 [0.10, 2.10]	0.85 [0.35, 1.35]	1.20 [−0.30, 2.70]

## Data Availability

The original contributions presented in this study are included in the article. Further inquiries can be directed to the corresponding author.

## References

[1] Fariña R., Canto L., Gunckel R., Alister J.P., Uribe F. (2018). Temporomandibular Joint Ankylosis: Algorithm of Treatment. J. Craniofacial Surg..

[2] Anchlia S., Vyas S.M., Dayatar R.G., Domadia H.L., Nagavadiya V. (2019). Guidelines for Single-Stage Correction of TMJ Ankylosis, Facial Asymmetry and OSA in Adults. J. Maxillofac. Oral Surg..

[3] Miloro M., Ghali G.E., Larsen P.E., Waite P. (2022). Peterson’s Principles of Oral and Maxillofacial Surgery.

[4] Shira R.B., Moorthy A.P., Ffdrcsi F., Finch L.D., Bds C. (1983). Interpositional arthroplasty for ankylosis of the temporomandibular joint. Oral Surg. Oral Med. Oral Pathol..

[5] Brusati R., Raffaini M.J., Bozzetti A. (1990). The Temporalis Muscle Flap in Temporo-Mandibular Joint Surgery. J. Cranio-Maxillofac. Surg..

[6] Arakeri G., Kusanale A., Zaki G.A., Brennan P.A. (2012). Pathogenesis of post-traumatic ankylosis of the temporomandibular joint: A critical review. Br. J. Oral Maxillofac. Surg..

[7] Zhu S., Wang D., Yin Q., Hu J. (2013). Treatment guidelines for temporomandibular joint ankylosis with secondary dentofacial deformities in adults. J. Cranio-Maxillofac. Surg..

[8] Mercuri L.G., Ali F.A., Woolson R. (2008). Outcomes of Total Alloplastic Replacement with Periarticular Autogenous Fat Grafting for Management of Reankylosis of the Temporomandibular Joint. J. Oral Maxillofac. Surg..

[9] Chen S., He Y., An J.G., Zhang Y. (2019). Recurrence-Related Factors of Temporomandibular Joint Ankylosis: A 10-Year Experience. J. Oral Maxillofac. Surg..

[10] Nitesh M., Kumar S.N., Kumar D.N., Chandresh J., Preeti T., Kumar S.A. (2021). Temporomandibular joint ankylosis: A tertiary center-based epidemiological study. Natl. J. Maxillofac. Surg..

[11] Bhatt K., Roychoudhury A., Balakrishnan P. (2013). Temporomandibular joint ankylosis: Is hypercoagulable state of blood a predisposing factor?. Med. Hypotheses.

[12] Mekonnen D., Gizaw A., Kebede B. (2021). Temporomandibular Joint Ankylosis among Patients at Saint Paul’s Hospital Millennium Medical College, Ethiopia: A 9-Year Retrospective Study. Int. J. Dent..

[13] Anyanechi C.E. (2015). Temporomandibular joint ankylosis caused by condylar fractures: A retrospective analysis of cases at an urban teaching hospital in Nigeria. Int. J. Oral Maxillofac. Surg..

[14] Sharma V.K., Rattan V., Rai S., Malhi P. (2019). Quality of life assessment in temporomandibular joint ankylosis patients after interpositional arthroplasty: A prospective study. Int. J. Oral Maxillofac. Surg..

[15] Dimitroulis G. (2011). A critical review of interpositional grafts following temporomandibular joint discectomy with an overview of the dermis-fat graft. Int. J. Oral Maxillofac. Surg..

[16] Mani B., Balasubramaniam S., Balasubramanian S., Jayaraman B., Thirunavukkarasu R. (2020). Role of custom-made prosthesis for temporomandibular joint replacement in unilateral ankylosis—An evaluative study. Ann. Maxillofac. Surg..

[17] Khalil S., Khamis H., Kauke M., Iizuka T., Safi A.F. (2021). Three-Dimensional Osteosynthesis Plates for the Surgical Treatment of Mandibular Fractures. J. Craniofacial Surg..

[18] Rao J.D., Dar N., Sharma A., Sheorain A., Malhotra V., Arya V. (2017). Evaluation of the sternoclavicular graft for the reconstruction of temporomandibular joint after gap arthroplasty. Ann. Maxillofac. Surg..

[19] Al-Hudaid A., Aldialami A., Helmi J., Al-Wesabi M., Madfa A. (2017). Management of temporomandibular joint ankylosis in Yemeni children by metatarsal bone grafts. J. Oral Res..

[20] Liu X., Shen P., Zhang S., Yang C., Wang Y. (2015). Effectiveness of different surgical modalities in the management of temporomandibular joint ankylosis: A meta-analysis. Int. J. Clin. Exp. Med..

[21] Resnick C.M., Genuth J., Calabrese C.E., Taghinia A., Labow B.I., Padwa B.L. (2018). Temporomandibular Joint Ankylosis After Ramus Construction with Free Fibula Flaps in Children with Hemifacial Microsomia. J. Oral Maxillofac. Surg..

[22] Yang Y.T., Li Y.F., Jiang N., Bi R.Y., Zhu S.S. (2018). Grafts of autogenous coronoid process to reconstruct the mandibular condyle in children with unilateral ankylosis of the temporomandibular joint: Long-term effects on mandibular growth. Br. J. Oral Maxillofac. Surg..

[23] Roychoudhury A., Yadav P., Bhutia O., Mane R., Yadav R., Goswami D., Jose A. (2021). Alloplastic total joint replacement in management of temporomandibular joint ankylosis. J. Oral Biol. Craniofac. Res..

[24] Gerbino G., Sobrero F., Poelaert R., Borbon C., Ramieri G., Mommaerts M. (2025). Extended temporomandibular joint prostheses: A retrospective analysis of feasibility, outcomes, and complications. Int. J. Oral Maxillofac. Surg..

[25] Borbon C., Zavattero E., Ramieri G., Gerbino G. (2023). Management of failed autogenous tissues temporomandibular joint reconstruction: Case series and literature review. Front. Oral Maxillofac. Med..

[26] Vincent S.D., Mercuri L.G. (1998). Alloplastic temporomandibular joint reconstruction. Oral Maxillofac. Surg. Clin. N. Am..

[27] Gerbino G., Zavattero E., Berrone S., Ramieri G. (2016). One stage treatment of temporomandibular joint complete bony ankylosis using total joint replacement. J. Cranio-Maxillofac. Surg..

[28] Burgess M., Bowler M., Jones R., Hase M., Murdoch B. (2014). Improved outcomes after alloplastic TMJ replacement: Analysis of a multicenter study from Australia and New Zealand. J. Oral Maxillofac. Surg..

[29] Aagaard E., Thygesen T. (2014). A prospective, single-centre study on patient outcomes following temporomandibular joint replacement using a custom-made Biomet TMJ prosthesis. Int. J. Oral Maxillofac. Surg..

[30] Giannakopoulos H.E., Sinn D.P., Quinn P.D. (2012). Biomet microfixation temporomandibular joint replacement system: A 3-year follow-up study of patients treated during 1995 to 2005. J. Oral Maxillofac. Surg..

[31] Gerbino G., Zavattero E., Bosco G., Berrone S., Ramieri G. (2017). Temporomandibular joint reconstruction with stock and custom-made devices: Indications and results of a 14-year experience. J. Cranio-Maxillofac. Surg..

[32] Al-Moraissi E.A., El-Sharkawy T.M., Mounair R.M., El-Ghareeb T.I. (2015). A systematic review and meta-analysis of the clinical outcomes for various surgical modalities in the management of temporomandibular joint ankylosis. Int. J. Oral Maxillofac. Surg..

[33] Sawhney C.P.M.S. (1986). Bony ankylosis of the temporomandibular joint: Follow-up of 70 patients treated with arthroplasty and acrylic spacer interposition. Plast. Reconstr. Surg..

[34] Shmueli A. (2005). The visual analogy rating scale of health-related quality of life: An examination of end-digit preferences. Health Qual Life Outcomes.

[35] Alonso-Royo R., Sánchez-Torrelo C.M., Ibáñez-Vera A.J., Zagalaz-Anula N., Castellote-Caballero Y., Obrero-Gaitán E., Rodríguez-Almagro D., Lomas-Vega R. (2021). Validity and Reliability of the Helkimo Clinical Dysfunction Index for the Diagnosis of Temporomandibular Disorders. Diagnostics.

[36] House J.W., Brackmann D.E. (1985). Facial nerve grading system. Otolaryngol.—Head Neck Surg..

[37] Zieliński G., Gawda P. (2025). Defining Effect Size Standards in Temporomandibular Joint and Masticatory Muscle Research. Med. Sci. Monit..

[38] Wolford L.M., Rodrigues D.B., McPhillips A. (2010). Management of the infected temporomandibular joint total joint prosthesis. J. Oral Maxillofac. Surg..

[39] Kaban L.B., Perrott D.H., Fisher K. (1990). A Protocol for Management of Temporomandibular Joint Ankylosis. J. Oral Maxillofac. Surg..

[40] Bansal S., Verma D.K., Rai M., Sorake A., Kaur C. (2019). Gap Arthroplasty or Interpositional Arthroplasty for the Management of TMJ Ankylosis? A Prospective Randomized Comparative Multicenter Clinical Trial. J. Maxillofac. Oral Surg..

[41] Mittal N., Goyal M., Sardana D. (2022). Autogenous grafts for reconstruction arthroplasty in temporomandibular joint ankylosis: A systematic review and meta-analysis. Br. J. Oral Maxillofac. Surg..

[42] Kalita F., Arunkumar K.V. (2023). Temporomandibular joint re-ankylosis: A case report and literature review. J. Korean Assoc. Oral Maxillofac. Surg..

[43] Bénateau H., Chatellier A., Caillot A., Diep D., Kün-Darbois J.D., Veyssière A. (2016). L’ankylose temporo-mandibulaire. Rev. Stomatol. Chir. Maxillo-Faciale Chir. Orale.

[44] Mercuri L.G. (2013). The role of custom-made prosthesis for temporomandibular joint replacement. Rev. Esp. Cir. Oral Maxilofac..

[45] Haq J., Patel N., Weimer K., Matthews N.S. (2014). Single stage treatment of ankylosis of the temporomandibular joint using patient-specific total joint replacement and virtual surgical planning. Br. J. Oral Maxillofac. Surg..

[46] Movahed R., Teschke M., Wolford L.M. (2013). Protocol for concomitant temporomandibular joint custom-fitted total joint reconstruction and orthognathic surgery utilizing computer-assisted surgical simulation. J. Oral Maxillofac. Surg..

[47] Wolford L.M., Pitta M.C., Reiche-Fischel O., Franco P.F. (2003). TMJ concepts/techmedia custom-made TMJ total joint prosthesis: 5-year follow-up study. Int. J. Oral Maxillofac. Surg..

[48] Sidebottom A.J., Gruber E. (2013). One-year prospective outcome analysis and complications following total replacement of the temporomandibular joint with the TMJ Concepts system. Br. J. Oral Maxillofac. Surg..

[49] Driemel O., Braun S., Müller-Richter U.D.A., Behr M., Reichert T.E., Kunkel M., Reich R. (2009). Historical development of alloplastic temporomandibular joint replacement after 1945 and state of the art. Int. J. Oral Maxillofac. Surg..

[50] Beret M., Nicot R., Gutman L., Ferri J., Quality J.F. (2022). Quality of Life After Total Temporomandibular Joint Prothesis Surgery. Quality of life after total temporomandibular joint prothesis surgery. Life After Total Temporo-mandibular Joint Prothesis Surgery. J. Craniofacial Surg..

[51] De Meurechy N.K.G., Loos P.J., Mommaerts M.Y. (2019). Postoperative Physiotherapy After Open Temporomandibular Joint Surgery: A 3-Step Program. J. Oral Maxillofac. Surg..

[52] Saini R.S., Ibrahim M., Khader M.A., Kanji M.A., Mosaddad S.A., Heboyan A. (2024). The role of physiotherapy interventions in the management of temporomandibular joint ankylosis: A systematic review and meta-analysis: Running title: Physiotherapy in TMJ ankylosis. Head Face Med..

[53] Yadav P., Roychoudhury A., Bhutia O. (2021). Strategies to reduce re-ankylosis in temporomandibular joint ankylosis patients. Br. J. Oral Maxillofac. Surg..

[54] Roychoudhury A., Yadav P., Alagarsamy R., Bhutia O., Goswami D. (2021). Outcome of Stock Total Joint Replacement with Fat Grafting in Adult Temporomandibular Joint Ankylosis Patients. J. Oral Maxillofac. Surg..

[55] Wolford L., Movahed R., Teschke M., Fimmers R., Havard D., Schneiderman E. (2016). Temporomandibular joint ankylosis can be successfully treated with TMJ concepts patient-fitted total joint prosthesis and autogenous fat grafts. J. Oral Maxillofac. Surg..

